# Genomic Analysis Reveals Potential Mechanisms Underlying Promotion of Tomato Plant Growth and Antagonism of Soilborne Pathogens by Bacillus amyloliquefaciens Ba13

**DOI:** 10.1128/Spectrum.01615-21

**Published:** 2021-11-10

**Authors:** Chenglong Ji, Meilin Zhang, Zirong Kong, Xue Chen, Xing Wang, Wei Ding, Hangxian Lai, Qiao Guo

**Affiliations:** a College of Natural Resources and Environment, Northwest A&F University, Yangling, China; b Laboratory of Natural Products Pesticide, College of Plant Protection, Southwest University, Chongqing, China; University of Minnesota

**Keywords:** genomic analysis, *Bacillus amyloliquefaciens*, plant growth promotion, antipathogenic activity

## Abstract

Bacillus amyloliquefaciens Ba13 is a plant beneficial bacterium isolated from loessial soil with notable biological activity. This study clarified potential mechanisms underlying the plant growth-promoting and antipathogenic effects of strain Ba13. A pot experiment was used to verify the plant growth-promoting effects of strain Ba13 on tomato, and the antipathogenic activity was tested in petri dishes. The underlying mechanisms were explored based on whole-genome sequencing of strain Ba13 and liquid chromatography-tandem mass spectrometry (LC-MS/MS) detection of plant hormones and biosynthetic intermediates. The results showed that exposure to strain Ba13 promoted tomato plant growth significantly. Compared with control treatment, bacterial treatment increased plant height and fresh weight by 10.98% and 20.15%, respectively, at 28 days after inoculation. Strain Ba13 exhibited antagonistic activity against all eight plant pathogens tested. The 3,861,210-bp genome of strain Ba13 was predicted to encode antibiotics (e.g., surfactin, bacillaene, bacillomycin D, bacilysin, and bacillibactin) and volatile gaseous compounds (e.g., 2,3-butanediol and acetoin). Genes were also predicted to encode extracellular phytase and β-glucanase that are secreted through the secretory (Sec) system. Strain Ba13 could synthesize indole-3-acetic acid through the indole-3-pyruvic acid pathway. The results of this study indicate that *B. amyloliquefaciens* Ba13 has multiple effects on tomato plants and associated microorganisms, directly or indirectly promoting plant growth and controlling plant diseases.

**IMPORTANCE** Microbial agents are considered the optimal alternative for chemical agents. Exploring the mechanisms underlying the beneficial effects of microbial agents is essential for rational applications in the field. In this study, we report a functional bacterial strain, Bacillus amyloliquefaciens Ba13, which exhibited plant growth-promoting and antipathogenic effects. The whole genome of strain Ba13 was sequenced, and functional genes of interest were predicted. Strain Ba13 could synthesize indole-3-acetic acid through the indole-3-pyruvic acid pathway.

## INTRODUCTION

The application of agrochemicals in soil and plants has known and potential risks to the environment and even humans (1). Agricultural chemicals and inorganic fertilizers are extensively used to prevent plant disease infestation and to promote plant growth. Frequently, agricultural chemicals and inorganic fertilizer application can alter soil microbial community structure (2) and cause antibiotic resistance gene contamination (3), water eutrophication (4, 5), and air pollution (6). Many studies have demonstrated that the rational use of microbial agents not only has favorable plant disease control (7, 8) and growth promotion effects (9) but also enhances environmental protection (10). Consequently, microbial agents are considered the optimal alternative for chemical agents (11).

*Bacillus* is a common group of plant-associated bacteria in soil (12). Generally, *Bacillus* can synthesize and secrete abundant and diverse bioactive substances (13). Owing to their sporulation ability, bacteria in the genus can be formulated into bacteria-based products that are stable for a long time and resistant to adverse environmental conditions (14). Numerous *Bacillus* species, including *B. amyloliquefaciens* (15), B. megaterium (16), and B. licheniformis (17), have been used to control plant disease and promote plant growth (11, 18). As a member of the B. subtilis species complex (19), *B. amyloliquefaciens* has gene clusters that encode multiple secondary metabolites with antipathogenic properties. *B. amyloliquefaciens* can also promote plant growth (20), with DSM7 (21) and SQR9 (22) being the notable strains.

The secretion of biosynthetic plant hormones is considered one of the major mechanisms via which beneficial bacteria promote plant growth (23, 24). Indole-3-acetic acid (IAA) enhances overall growth in plants by stimulating cell elongation, division, and differentiation (25). Therefore, bacteria capable of producing IAA may be effective plant growth promoters (22, 26). However, the IAA biosynthetic pathway in bacteria is not uniform. Three tryptophan (TRP)-dependent pathways, whose intermediate products are indole-3-pyruvic acid (IPyA), indole-3-acetamide (IAM), and tryptamine (TAM), have been reported and studied comprehensively (27–29). In the case of *B. amyloliquefaciens*, strain SQR9 could reportedly synthesize IAA through the IPyA pathway (22), and strain FZB42 could synthesize IAA through a TRP-dependent pathway (30). Other plant growth-promoting mechanisms have also been reported in bacteria that secrete extracellular enzymes to dissolve soil organic phosphorus (31), organic acids to dissolve mineral potassium (32), volatile organic compounds (VOCs) to support plant growth (33), and 1-aminocyclopropane-1-carboxylic acid deaminase to degrade ethylene (34).

The secondary metabolites synthesized by beneficial bacteria, including *Bacillus*, play a vital role in plant pathogen resistance. Various antibiotics, such as lipopeptides (surfactin, bacillomycin D, and fengycin), polyketides (macrolactin, bacillaene, and difficidin), and dipeptides (bacilysin), directly inhibit gene expression or alter pathogen cell membrane structures (35). Moreover, glucanase inhibits the growth of fungal hyphae directly by degrading the cell wall (36), whereas bacterial siderophores bind the limited iron resources in the soil environment and competitively inhibit the growth and reproduction of pathogens (37). In addition, surfactin and VOCs induce systemic resistance in plants and improve plant resistance to disease (38).

Previously, it has been reported that preinoculation of *B. amyloliquefaciens* Ba13 into soil could increase shoot biomass, improve rhizosphere microecology, and induce plant systemic resistance under tomato yellow leaf curl virus infection (39). However, it is still unclear whether the improvement in shoot biomass is due to the control of tomato yellow leaf curl virus disease or the promotion of plant growth by strain Ba13 and how the bacterial agent influences rhizosphere microecology and plant systemic resistance. Therefore, in the present study, the ability of *B. amyloliquefaciens* Ba13 to promote tomato plant growth and antagonize soilborne pathogens was verified using pot and petri dish experiments, respectively. Subsequently, the underlying mechanisms of plant growth promotion and antipathogenic properties of *B. amyloliquefaciens* Ba13 were explored by whole-genome sequencing and genomic analysis. In addition, the IAA biosynthesis capacity of strain Ba13 and the associated pathway were preliminarily verified.

## RESULTS

### Plant growth-promoting effect of *B. amyloliquefaciens* Ba13.

**FIG 1:**
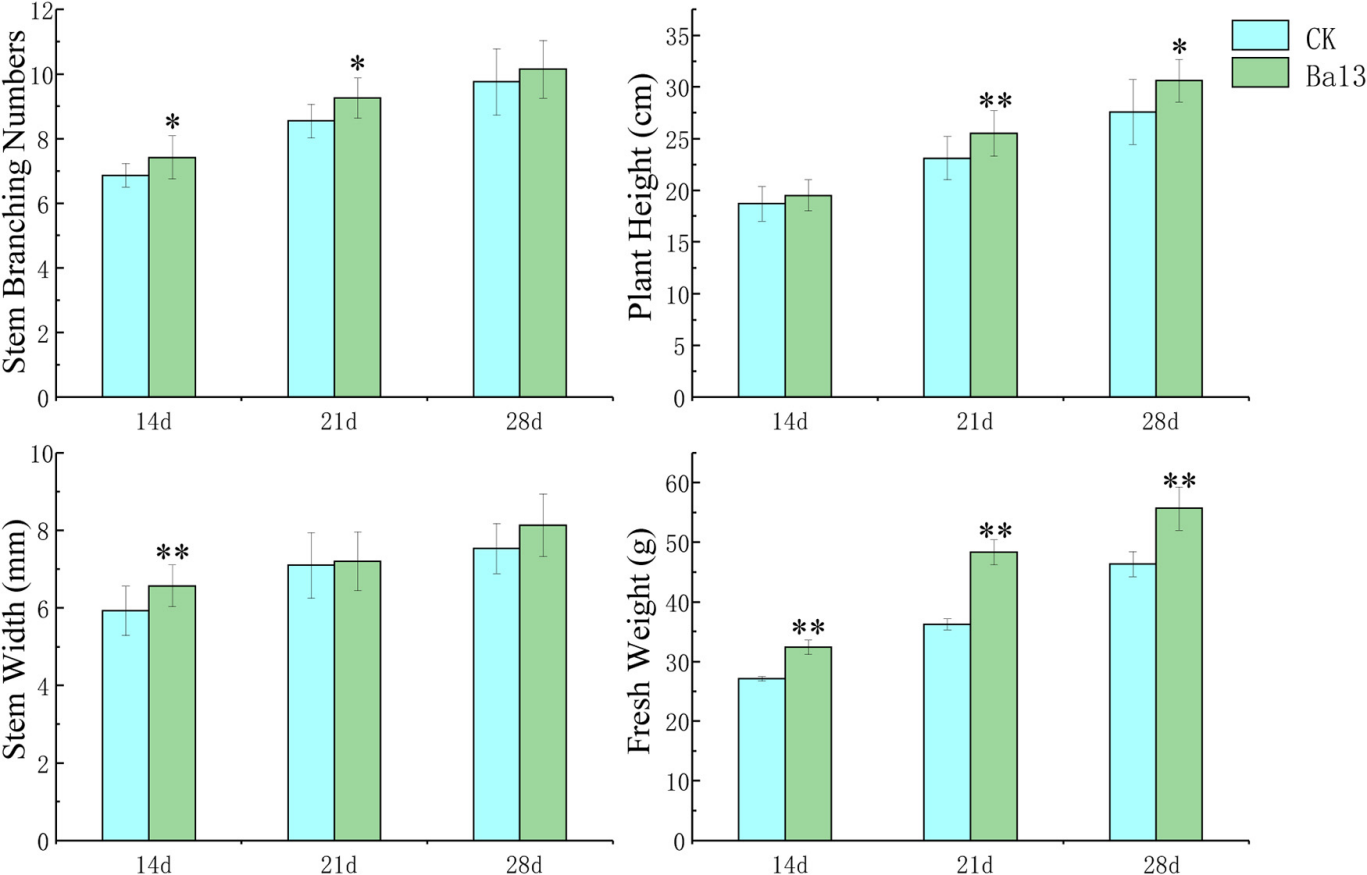
Effects of *B. amyloliquefaciens* Ba13 on plant growth of tomato in pots. Histograms show the difference in plant growth parameters between treatments at three growth stages. Data represent mean ± standard deviation; CK, control check (a control treatment without strain Ba13); *, *P* < 0.05; **, *P* < 0.01.

Compared with the control group, the growth parameters of tomato plants treated with strain Ba13 were generally higher across different growth stages (Fig. 1). On the 14th day, stem branching number, stem width, and fresh weight of the Ba13-treated group increased by 7.69%, 10.89%, and 19.52%, respectively. On the 21st day, plant height, stem branching number, and fresh weight of the Ba13-treated group increased by 10.36%, 7.21%, and 33.30%, respectively. On the 28th day, plant height increased by 10.98% and fresh weight increased by 20.15% in the Ba13-treated group.

### Antipathogenic activity of *B. amyloliquefaciens* Ba13.

**FIG 2:**
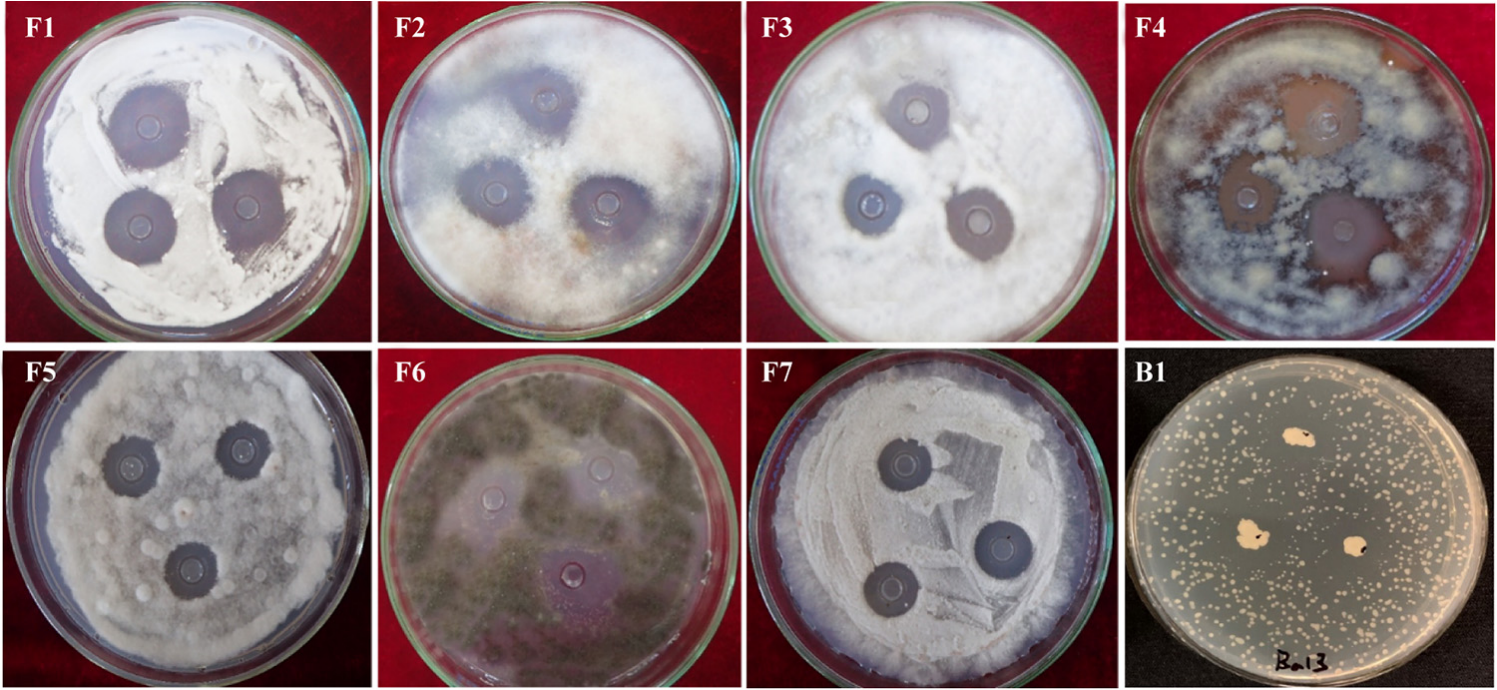
Antagonistic activity of *B. amyloliquefaciens* Ba13 against fungal and bacterial pathogens grown on potato dextrose agar in petri dishes; F1, Verticillium dahliae; F2, Fusarium solani; F3, Fusarium oxysporum f. sp*. cucumerinum*; F4, Exserohilum turcicum; F5, Fusarium oxysporum f. sp*. melonis*; F6, Alternaria dauci; F7, Sclerotinia sclerotiorum; B1, Ralstonia solanacearum.

Strain Ba13 exhibited antagonistic activity against all nine plant pathogens tested (Fig. 2). The greatest antagonistic effects were observed on pathogens Verticillium dahliae, Fusarium solani, Exserohilum turcicum, and Alternaria dauci based on the diameter of the growth inhibitory zone (>6 mm). Relatively low effects were observed on other pathogens, with a diameter of the growth inhibitory zone of >4 mm.

### Identification of *B. amyloliquefaciens* Ba13.

**FIG 3:**
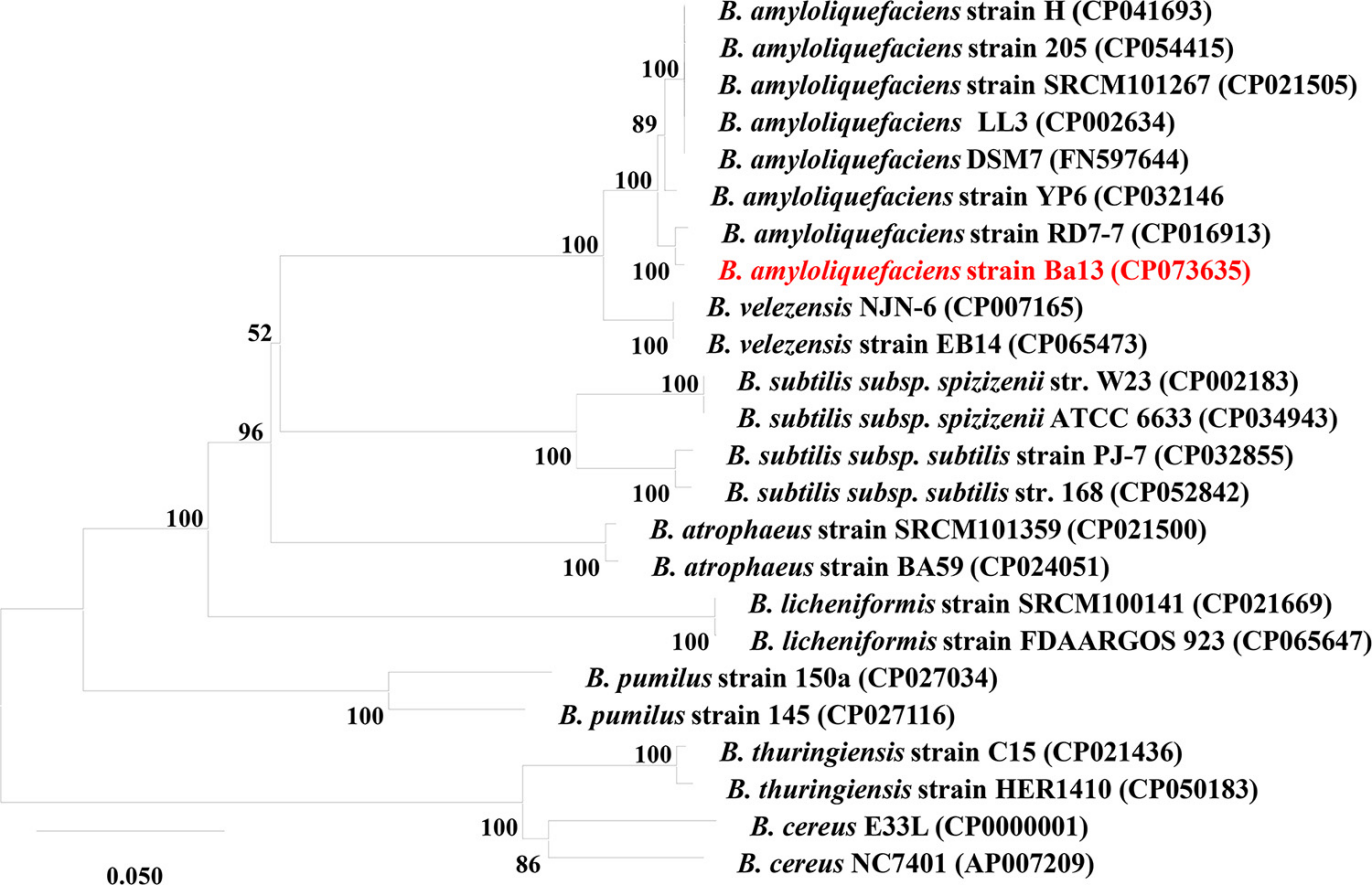
A phylogenetic tree constructed based on the *gyrB* gene sequences.

When growing on beef extract peptone agar, strain Ba13 formed irregular translucent colonies with a white to slightly yellow color and folded ridges. The colonies did not produce pigments (Fig. S1a in the supplemental material). Both agar and broth cultures emitted an odor similar to natto fermentation. Scanning electron microscopy (SEM) observation showed that the cells were stubby round rods without flagella (Fig. S1b). Phylogenetic analysis revealed that strain Ba13 was the most closely related to *B. amyloliquefaciens* RD7-7 (99.5% sequence similarity), and its close relatives were also *B. amyloliquefaciens* (Fig. 3). Accordingly, strain Ba13 was identified as *B. amyloliquefaciens*.

### Genomic information of *B. amyloliquefaciens* Ba13.

**FIG 4:**
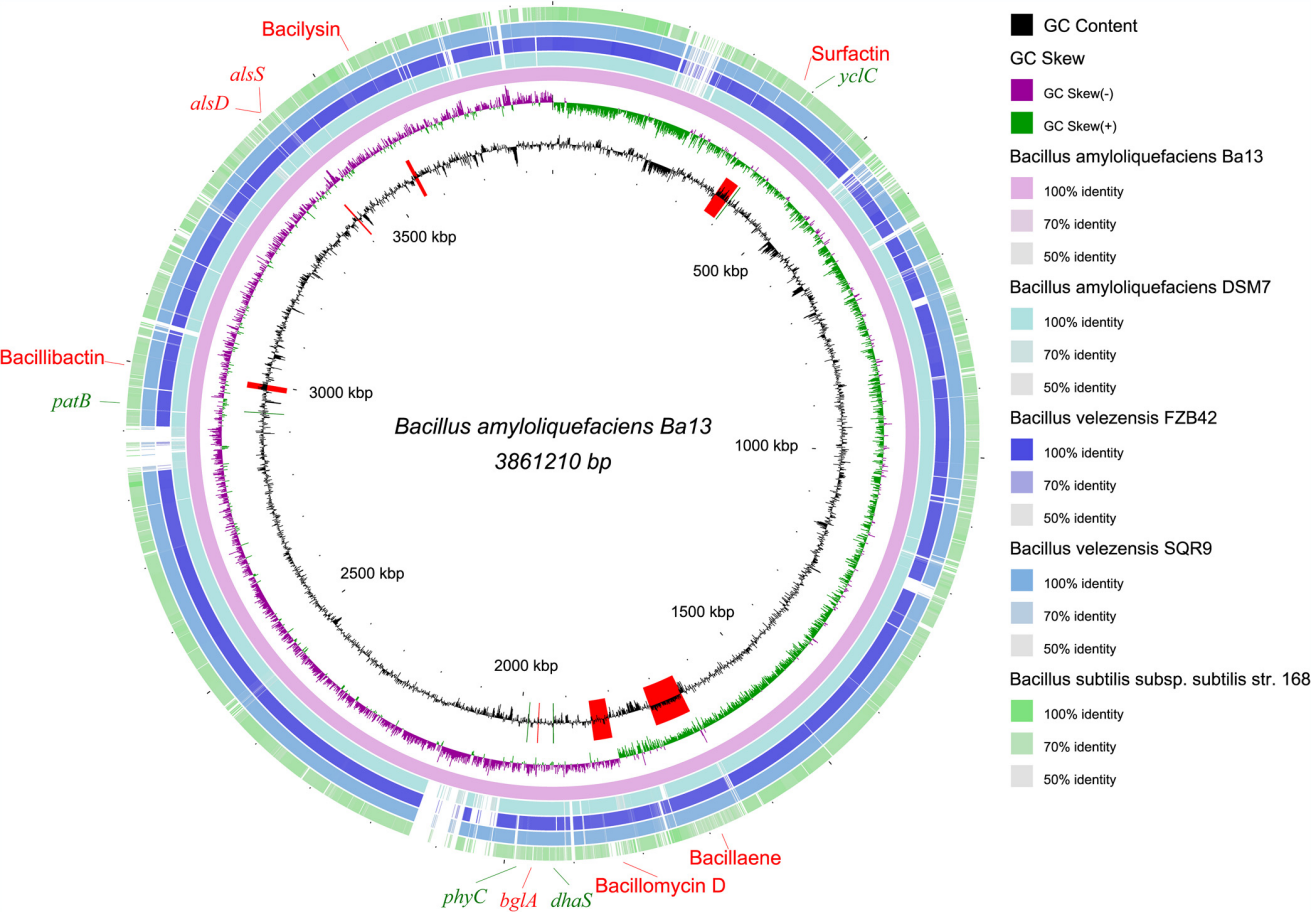
Comparative genomic circle map of *B. amyloliquefaciens* Ba13 constructed using BRIG v0.95. The features are as follows (from center to outside): circle 1 is genome size; circle 2 is GC content; circle 3 is GC skew; circles 4 to 8 are the comparative genomic maps of *Bacillus* strains DSM7, FZB42, SQR9, and 168, respectively, with the Ba13 genome as the reference; outside the circle are the locations of genes and gene clusters involved in growth promotion and antimicrobial activities. Surfactin, bacillaene, bacillomycin D, bacillibactin, and bacilysin denote five antibiotic gene clusters. *patB*, *yclC*, and *dhaS* indicate genes involved in IAA biosynthesis. *phyC* represents the gene related to extracellular phytase biosynthesis. *bglA* indicates the gene related to endo β-1,3-1,4 glucanase biosynthesis. *alsD* and *alsS* represent genes involved in the biosynthesis of 2,3-butanediol and acetoin.

**FIG 5:**
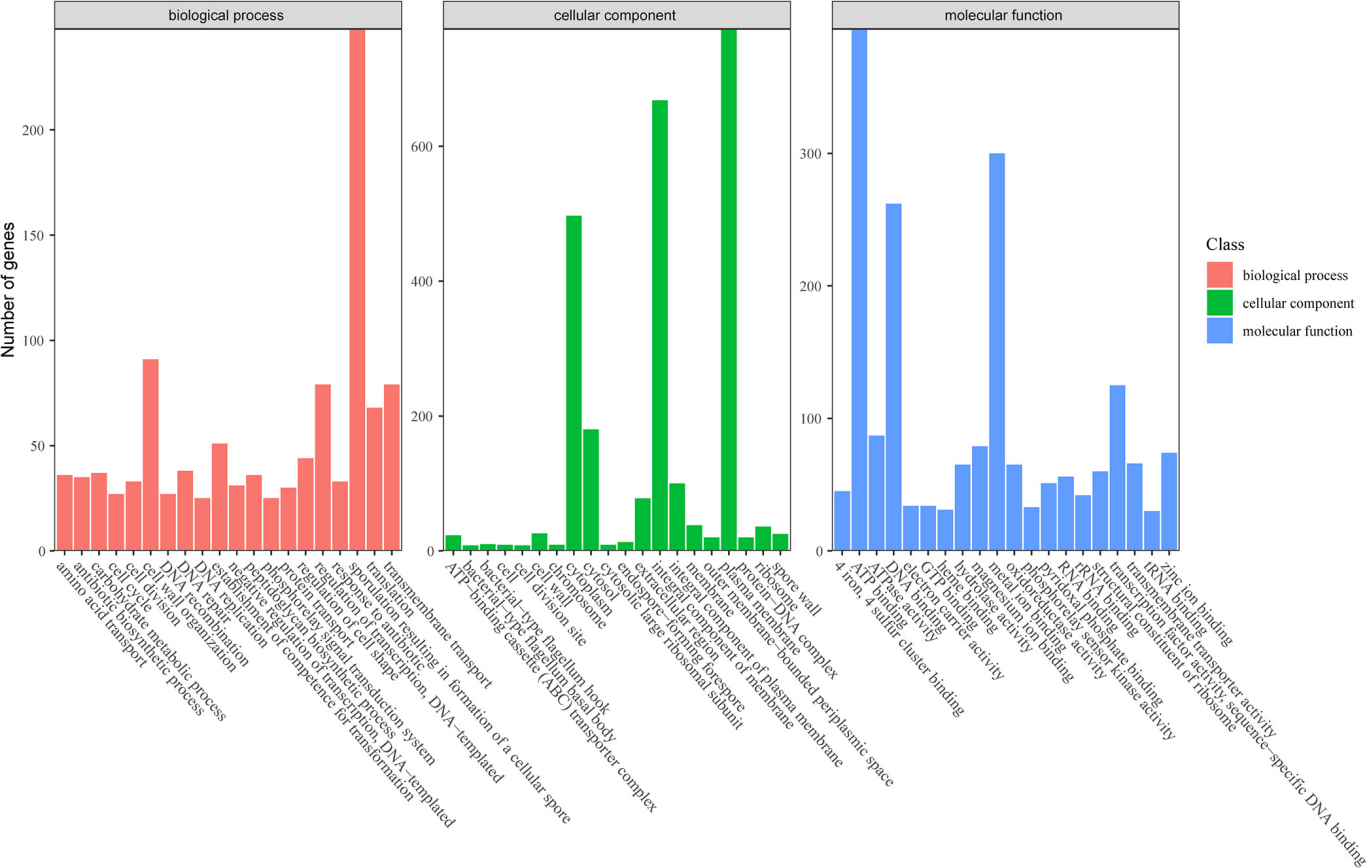
Annotated map of gene function classification in the Gene Ontology database for the whole genome of *B. amyloliquefaciens* Ba13.

A complete circular genome of strain Ba13 was assembled with a full length of 3,861,210 bp, a GC content of 46.2%, and a mean sequencing depth of 308.16× (Fig. 4). The genome contained 4,019 genes, including 3,825 coding DNA sequence (CDS) genes, 87 tRNA genes, 27 rRNA genes, and a transfer-messenger RNA (tmRNA) gene. Subsequently, 71.98% of the genes were annotated into the Gene Ontology database, and the top 20 secondary categories of the annotated genes were selected from three categories (cell composition, molecular function, and biological process) to describe the functional focus of the genes in the whole genome (Fig. 5). There were 4,664 genes annotated in the molecular function process, including 394 ATP-binding-related genes, 300 metal ion-binding-related genes, and 262 DNA-binding-related genes. In the cellular component process, 2,752 genes were annotated, including 774 related to plasma membrane, 668 related to integral component of membrane, and 497 related to cytoplasm. There were 3,299 genes annotated in biological process, with 248 related to sporulation resulting in the formation of a cellular spore, 91 related to cell wall organization, and 78 genes related to DNA-templated regulation of transcription.

### Prediction of genes with growth promotion and antipathogenic functions.

*Secondary metabolite prediction.* Ten secondary metabolite gene clusters were predicted in the whole genome of strain Ba13 (Table 1). Five of the gene clusters encode surfactin (region 2), bacillaene (region 5), bacillomycin D (region 6), bacillibactin (region 9), and bacilysin (region 10). The remaining five gene clusters have no predicted products; however, one of them (region 1) exhibits low similarity with the locillomycin biosynthesis gene cluster, and two unknown secretion clusters (region 4 and region 7) synthesize terpene.

**TABLE 1:** Ten predicted gene clusters of secondary metabolites in the genome of *B. amyloliquefaciens* Ba13

Region	Cluster category	Location	Size (bp)	Types of metabolites	Metabolite	Function
1	NRPS/PKS	193249–277040	83,791	Polyketides		
2	NRPS	347177–411489	64,312	Lipopeptides	Surfactin	Antibacterial, antifungal
3	PKS-like	931932–973176	41,244	Saccharide		
4	Terpene	1058136–1078548	20,412	Terpene		
5	NRPS/PKS	1630195–1739391	109,196	Polyketides	Bacillaene	Antibacterial
6	NRPS	1791572–1886211	94,639	Lipopeptides	Bacillomycin D	Antifungal, antibacterial
7	Terpene	1920035–1941918	21,883	Terpene		
8	T3PKS	1996351–2037451	41,100	Polyketides		
9	NRPS	2968187–3017897	49,710	Lipopeptides	Bacillibactin	Siderophores
10	NRPS	3538651–3580069	41,418	Dipeptide	Bacilysin	Antibacterial, algicidal activity

*Secretory protein prediction.* A total of 522 sequences with signal peptides were predicted, including 159 transmembrane proteins, 79 membrane surface lipoproteins, 28 intramembrane proteins, five twin-arginine translocation (TAT)-secreted proteins, and 251 Sec-secreted proteins. Out of the 238 transmembrane proteins and membrane surface lipoproteins, 221 were annotated in the UniProt database. The following functional proteins were found on the cell membrane: (i) signal peptidase I and lipoprotein signal peptidase (signal peptidase II), which guide the classical protein secretion pathway; (ii) translocase protein TatCd, an important component of the TAT secretion pathway; (iii) components of the Sec secretion pathway, including protein translocase subunits (*SecY*, *SecDF*, and *SecG*), membrane protein insertase (*MisCA* and *MisCB*/*YidC*), and ATP-dependent zinc metalloprotease (*FtsH*); (iv) some antibiotic-binding and transporter proteins that may convey antibiotic resistance to strain Ba13 against bacitracin, linearmycin, penicillin, and petrobactin; (v) transport and binding proteins of zinc, copper, manganese, magnesium, iron, and ferrous iron; and (vi) binding and transporter proteins of iron-protein complex, protein, carbohydrate, amino acid, and polypeptide. Out of the 256 predicted extracellular proteins, only 166 were annotated in the UniProt database with a definite or presumed function. Most (124) of the 166 proteins were enzymatic substances, including 24 oxidoreductases, 22 transferases, 69 hydrolases, and 5 lyases. There were pectin lyases, peptidases, and other enzymes that are related to the decomposition and utilization of organic matter, phytase (a plant growth-promoting enzyme capable of decomposing the main component of soil organic phosphorus), and endo β-1,3-1,4 glucanase, which has the ability to degrade fungal cell walls.

*Prediction of plant hormone biosynthesis*. The genes of strain Ba13 were annotated in the KEGG database. KEGG pathway enrichment analysis revealed that strain Ba13 could synthesize TRP and indole in the TRP biosynthetic pathway (Fig. S2), both of which are substrates of IAA biosynthesis. In the TRP metabolic pathway (Fig. S3), genes were enriched in the common IAA biosynthetic pathways; yet, all four pathways were found to be incomplete. However, searching the genome of strain Ba13 revealed the presence of *patB*, *yclC* (phenolic acid decarboxylase), and *dhaS* genes, which constitute a potential complete IpyA pathway of IAA biosynthesis, Therefore, at the gene level, strain Ba13 can synthesize IAA through the IpyA pathway. In addition, strain Ba13 may synthesize IAA through the indole biosynthetic pathway, whose mechanism has not yet been studied comprehensively.

*Other genes with growth-promoting and antipathogenic functions.* Based on the annotation results, the genome of strain Ba13 was observed to harbor VOC biosynthesis genes (*AlsS* and *AlsD*). The genes encode enzymes related to the biosynthesis of 2,3-butanediol and acetoin, which could promote plant growth following volatilization into the surrounding atmosphere (33).

### Levels of plant hormones and biosynthetic intermediates.

In the culture supernatant of strain Ba13, a red color reaction with Pilet and Chollet (PC) reagent occurred (Fig. S4), indicating that strain Ba13 could synthesize IAA from TRP. Subsequently, the levels of plant hormones and their biosynthetic intermediates in the cell-free culture supernatant of strain Ba13 were quantified using liquid chromatography-tandem mass spectrometry (LC-MS/MS; Table 2). The concentration of IAA was 6.68E−11 mol/liter. Among the other compounds detected, TRP had the highest contents (80.9% of the total), and its concentration reached 1.62E−08 mol/liter. There were several compounds involved in the IpyA biosynthetic pathway, namely, indole-3-acetaldehyde (IAD) at a concentration of 2.45E−09 mol/liter (12.3% of the total) and indole-3-pyruvic acid (IPA) at a concentration of 7.77E−10 mol/liter (3.9% of the total). As for the other IAA biosynthetic pathways, tryptamine (TRA) was detected at a concentration of 2.14E−10 mol/liter (1.1% of the total).

**TABLE 2:** Levels of plant hormones and biosynthetic intermediates in the cell-free culture supernatant of *B. amyloliquefaciens* Ba13

Compounds	Simple name	Density (mol/liter)	Percentage (%)
l-Tryptophan	TRP	1.62E−08	0.809
Indole-3-acetaldehyde	IAD	2.45E−09	0.123
Indole-3-pyruvic acid	IPA	7.77E−10	0.039
Tryptamine	TRA	2.14E−10	0.011
Indole-3-carboxylic acid	ICA	2.03E−10	0.010
Indole-3-acetic acid	IAA	6.68E−11	0.003
Indole-3-lactic acid	ILA	6.05E−11	0.003
Indole-3-carboxaldehyde	ICAld	4.81E−11	0.002

## DISCUSSION

In the present study, we report the plant growth promotion and antipathogenic activities of a functional bacterial strain, *B. amyloliquefaciens* Ba13. Additionally, we explored the potential mechanisms underlying the effects of strain Ba13 by whole-genome sequencing, KEGG pathway enrichment analysis, prediction of secreted proteins and secondary metabolites, and LC-MS/MS analysis. The results indicated that strain Ba13 could synthesize IAA and various antibiotics, produce growth-promoting VOCs (2,3-butanediol and acetoin), and secrete growth-promoting extracellular proteins (β-glucanase and phytase) through the Sec system.

The *B. amyloliquefaciens* group in the genus *Bacillus* contains three related species, including *B. amyloliquefaciens*, *B. velezensis*, and *B. siamensis*. *B. amyloliquefaciens* (40) and *B. velezensis* (41) have been renamed and restored several times in the past 20 years (42). Sometimes, it is impossible to distinguish the two species based on 16S rRNA gene sequencing (43), and further similarity analyses should be performed based on the *gyrB* gene (44). Therefore, we constructed a phylogenetic tree using the *gyrB* sequence annotated in the whole genome of strain Ba13. Strain Ba13 was identified as *B. amyloliquefaciens* based on the reliable bootstrap values and its close evolutionary distance from multiple strains of *B. amyloliquefaciens* in combination with colony and cell morphologies.

In the pot experiment, strain Ba13 exhibited a capacity to promote tomato plant growth. Exposure to strain Ba13 increased plant height and fresh weight markedly, which was partly related to the biosynthesis and secretion of IAA by the bacterial agent (30). Pathway enrichment analysis of the whole genome revealed that strain Ba13 has complete biosynthetic pathways of TRP and indole. Combined with a previous study on IAA biosynthesis in *B. amyloliquefaciens* (22), we found the presence of several genes (*patB*, *yclC*, and *dhaS*) that encode enzymes related to the IPyA biosynthetic pathway in strain Ba13. The result demonstrates that strain Ba13 has the capacity to synthesize IAA at the gene level. Furthermore, IAA was detected in the 48-h-old culture of strain Ba13 supplemented with additional TRP, which corroborated the prediction results of IAA biosynthesis. In addition, IAA together with TRP, IAD, IPA, and TRA were detected in the bacterial culture without additional TRP by LC-MS/MS.

TRP is the main precursor of IAA biosynthesis (45, 46). IAD and IPA are intermediate products of the IPyA biosynthetic pathway (47). TRA is the product of TRP directly catalyzed by decarboxylase (encoded by the *yclC* gene) and an intermediate product of the TRA pathway (48). Therefore, we assume that strain Ba13 independently synthesizes IAA and its substrates; it produces IAA mainly through the IPyA pathway to promote plant growth. In addition, the 2,3-butanediol and acetoin synthesized by *B. amyloliquefaciens* can facilitate plant growth when volatilized into the surrounding atmosphere, and the associated synthase genes also appear in the genome of strain Ba13 (33, 49). The prediction results of secretory proteins indicated that strain Ba13 could secrete phytase through the Sec system. Phytase can decompose the major component of soil organic phosphorus that is not readily absorbed by plants (31), and phytase secretion is one of the potential plant growth promotion mechanisms of strain Ba13.

**FIG 6:**
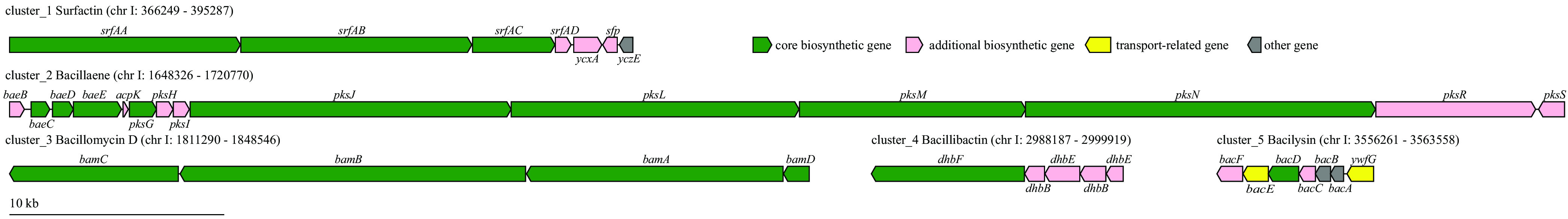
Gene cluster structures of five secondary metabolites in the genome of *B. amyloliquefaciens* Ba13. Clusters 1 to 5 represent the gene clusters of surfactin, bacillaene, bacillomycin D, bacillibactin, and bacilysin, respectively.

In the antagonistic experiment, the presence of strain Ba13 inhibited the growth of all fungal and bacterial pathogens tested to different degrees. It has also been reported previously that following the application of strain Ba13 into soil, the rhizosphere microbial community structure was altered, with an increase in the proportion of soil bacteria and a reduction in the number of pathogenic fungi (39). Secretion of antibiotics is one of the main causes of antagonism between microorganisms. In the present study, 10 secondary metabolite gene clusters were predicted in the genome of strain Ba13, 5 of which were identified as surfactin, bacillaene, bacillomycin D, bacillibactin, and bacilysin gene clusters with high similarity (Fig. 6). These five antibiotics have been reported to have antimicrobial activity; however, their mechanisms of antagonism are different.

Surfactin and bacillomycin D are cyclic lipopeptides biosynthesized by nonribosomal peptide synthetase (NRPS). Surfactin is a powerful biosurfactant with dose-dependent effects (50) and destroys cell membrane integrity through surface activity (51, 52) and exhibits antibacterial activity (53). Bacillomycin D has strong antifungal activity (54) depending on membrane permeability properties (55). In addition, surfactin may play a synergistic role in the promotion of the antifungal activity of bacillomycin D. The synergistic effects of surfactin reportedly enhance the antibiotic activity of iturin A (56). The antibiotic activity of bacillomycin D, a member of the iturin family, could be also enhanced by iturin A. Bacilysin is a dipeptide synthesized by NRPS, which exhibits algicidal (57) and antibacterial activity (58). Antimicrobial activity of bacilysin depends on the anticapsin moiety, which is released by peptidases. Intracellular anticapsin then blocks glucosamine synthetase, resulting in protoplasting and lysis of bacteria and fungi (58). Bacillaene is a polyene synthesized by *trans*-acyltransferase polyketide synthases, and its antibacterial activity is achieved by inhibiting prokaryotic protein biosynthesis (59).

Unlike the above-mentioned secondary metabolites, bacillibactin has unique biological functions and exhibits no direct toxicity to pathogens. It is an iron chelator identified in *Bacillus* (60), scavenging iron from environmental stocks and delivering it to the cell (61). In a previous study (39), the application of strain Ba13 increased the number of *B. velezensis* B3 in the rhizosphere, rhizoplane, and even the root endosphere of tomato considerably. Such effects could be attributed to the specific siderophore (bacillibactin) secreted by strain Ba13. Subsequently, we predicted the secondary metabolites of all strains in the phylogenetic tree. According to the results, *Bacillus* species with relatively close evolutionary distances, such as *B. amyloliquefaciens*, *B. velezensis*, B. subtilis subsp*. spizizenii*, B. subtilis subsp*. subtilis*, B. atrophaeus, and B. licheniformis, could all synthesize bacillibactin through gene clusters with 100% similarity. However, as the evolutionary distance increased, the similarity between the siderophore biosynthesis gene clusters and bacillibactin decreased gradually, which might lead to a structural change in the biosynthetic siderophore.

Structural characteristics facilitate the identification of siderophores and influence their absorption activities (62). The strain Ba13 applied to the soil potentially secreted high bacillibactin amounts, which were absorbed and utilized by the homologous receptors of other bacteria (e.g., *B. velezensis* B3) that were closely related to strain Ba13 based on evolutionary distance (63, 64). In addition, siderophores in the soil compete for soluble Fe^3+^ against microorganisms with no homologous receptors (37), which could partly be the reason for the increase in the proportion of soil bacteria and the decrease in the proportion of soil fungi following the application of strain Ba13. In addition, the genome of strain Ba13 contains two terpene gene clusters of unknown products that may encode signal molecules essential for plant growth (65) and three NRPS/polyketide synthase (PKS) gene clusters of unknown products. Among the other antipathogenic active compounds that do not belong to antibiotics, β-1,3-1,4 glucanase secreted by the Sec system also has the ability to hydrolyze fungal cell walls with antifungal activity (36, 66).

Induced systemic resistance (ISR) refers to the enhancement of disease defense due to local stimulation of nonpathogenic microorganisms (67). Although ISR is not a direct antipathogen effect caused by microorganisms, it plays an equally indispensable role in plant disease defense. Several strains of *Bacillus*, including strain Ba13, have been reported to induce ISR in plants (14, 39). According to the detection results of plant root secretions from *Bacillus* (68), it is speculated that ISR is triggered mainly by surfactin, VOCs, or other unidentified secondary metabolites (35). In fact, after purification, surfactin, 2,3-butanediol, and acetoin have been reported to induce and activate plant ISR (33, 38).

**FIG 7:**
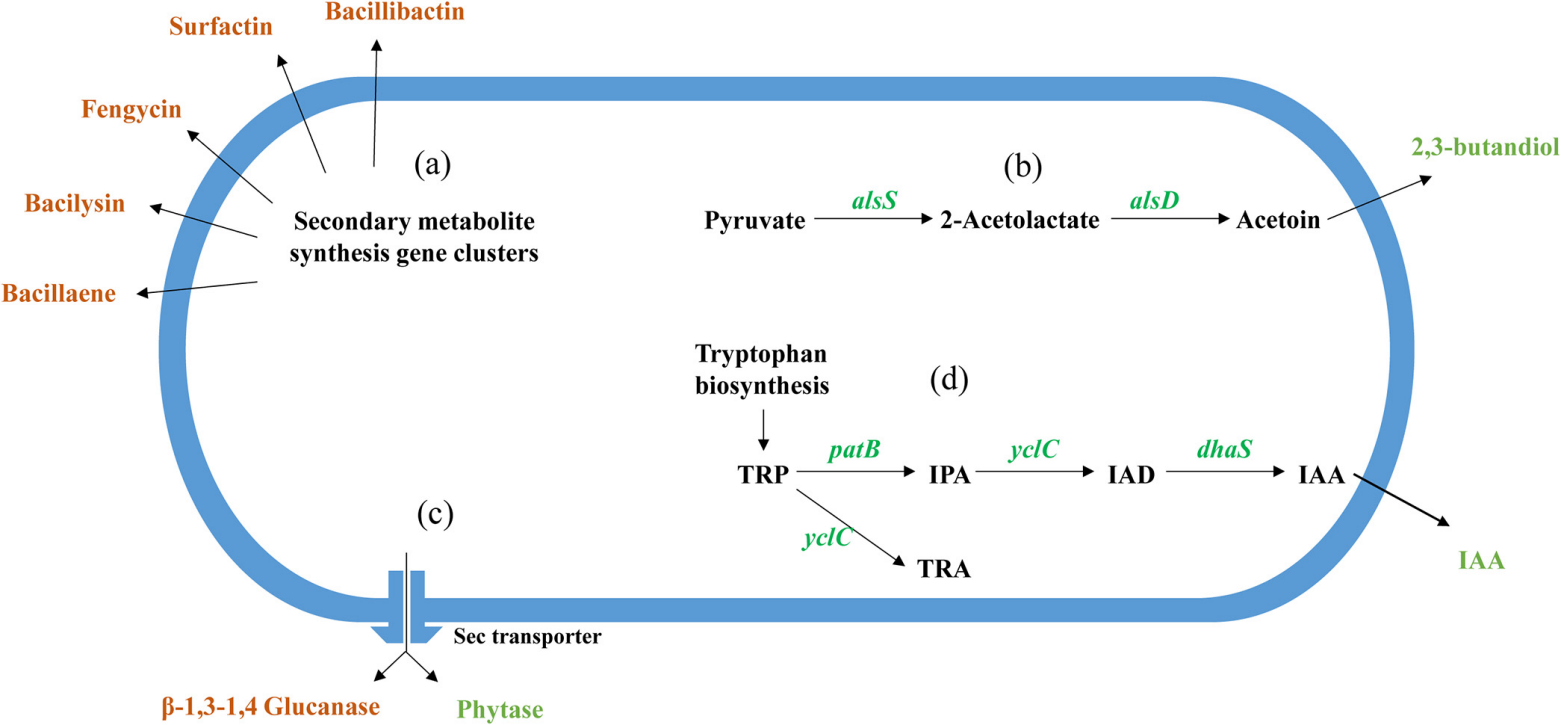
Schematic of the mechanisms underlying growth-promoting and antipathogenic effects of *B. amyloliquefaciens* Ba13. The brown color indicates the compounds associated with interactions between bacteria, and the green color represents the compounds related to plant growth promotion. (a) Secondary metabolites predicted in the genome. (b) Biosynthetic pathways of volatile gaseous compounds that promote plant growth. (c) Extracellular enzymes secreted by the Sec secretion system. (d) Indole-3-acetic acid (IAA) biosynthetic pathway; TRP, tryptophan; IPA, indole-3-pyruvic acid; IAD, indole-3-acetaldehyde; TRA, tryptamine.

In summary, the IAA, phytase, siderophores, antibiotics, and VOCs secreted by *B. amyloliquefaciens* Ba13 all act on plants or pathogens in different ways (Fig. 7), potentially promoting plant growth, inhibiting pathogenic growth, and improving plant resistance. Although it is not yet possible to accurately measure the relative contributions of each factor, the positive effects of strain Ba13 on plants are certainly directly or indirectly related to these factors (69). Based on the functional analysis and verification of strain Ba13, this study summarizes the known and potential plant growth-promoting and antipathogenic mechanisms of *B. amyloliquefaciens* Ba13. The results of the present study offer insights into the biological function of *Bacillus amylolysis* that could facilitate the development of known secondary metabolites or the exploration of unknown secondary metabolites and protein activators from this bacterial species.

## MATERIALS AND METHODS

### Test strains.

Bacillus amyloliquefaciens Ba13 was isolated from a loessial soil in Yangling, Shaanxi Province, China. Pure culture of strain Ba13 was preserved in the Resource Microbiology Laboratory, Northwest A&F University (Yangling, China). To examine the colony morphology of strain Ba13, the culture was inoculated on beef extract peptone agar plates (39) and observed after 72 h of culture at 37°C. For microscopic observation, cell cultures of strain Ba13 were fixed on a 7-mm circular coverslip with 2.5% glutaraldehyde solution, washed with phosphoric acid buffer (pH of 7.2), and dehydrated with a graded ethanol series. After critical point drying with liquid carbon dioxide, the micromorphology of strain Ba13 was observed under a Hitachi S-4800 scanning electron microscope (Hitachi, Japan).

Seven fungal pathogens were tested in the present study: F1, Verticillium dahliae, which causes *Verticillium* wilt of cotton; F2, Fusarium solani, which causes root rot of cucumber; F3, Fusarium oxysporum f. sp*. cucumerinum*, which causes Fusarium wilt of cucumber; F4, *Exserohilum turcicum*, which causes northern leaf blight of corn; F5, Fusarium oxysporum f. sp*. melonis*, which causes Fusarium wilt of melon; F6, *Alternaria dauci*, which causes early blight of tomato; and F7, Sclerotinia sclerotiorum, which causes stem rot disease in oilseed rape. Strains F1, F2, F3, F4, and F5 were obtained from the Resource Microbiology Laboratory, and strains F6 and F7 were provided by the College of Plant Protection, Northwest A&F University. One bacterial pathogen, B1, Ralstonia solanacearum, which causes bacterial wilt of tobacco, was provided by the Natural Products Pesticide Laboratory, Southwest University (Chongqing, China).

### Plant pot experiment.

The pot experiment was based on a random block design with two treatments, including a bacterial treatment with strain Ba13 and a control treatment without strain Ba13. Tomato (Lycopersicon esculentum cv. Jinpeng F1) seeds were purchased from Jinpeng Seed Co., Ltd. (Xi’an, China) and surface disinfected with 75% ethanol for 1 min. After five rinses with sterile pure water, the seeds were placed in a sterile dish with wet filter paper and incubated in the dark for germination. Then, the seedlings were transferred to the nursery substrate comprising peat, perlite, and vermiculite (2:1:1 [wt/wt/wt]). After true leaves emerged, the seedlings with consistent growth were selected and transplanted into plastic pots (height of 20 cm and diameter of 14 cm) containing 3 kg of air-dried loessial soil that was collected in Yangling. Each pot contained a single plant, and all pots were placed in a greenhouse with a mean temperature of 28°C and a daily12-h light cycle.

*B. amyloliquefaciens* Ba13 was cultured in beef extract peptone broth at 37°C and 180 rpm. The 48-h-old broth culture was diluted 100-fold (1 × 10^7^ CFU ml^−1^) and used to irrigate the plant roots (150 ml per pot). The control treatment received beef extract peptone broth diluted 100-fold without strain Ba13. Each treatment had 30 replicates. Plant growth was examined 14, 21, and 28 days after treatment. Five plants were randomly selected for destructive sampling. The fresh weight, plant height, stem width, and stem branch number of all sampled plants were measured. Data were analyzed using Excel 2016 (Microsoft Corp., Redmond, WA, USA) and PASW Statistics 18 (SPSS Inc., Chicago, IL, USA).

### Microbial antagonism experiment.

The Oxford cup method (inner diameter of 6 mm, outer diameter of 8 mm, height of 10 mm) was used for the antagonism experiment (70). All seven fungal pathogens were cultured on potato dextrose agar (PDA) plates for 72 h at 30°C. Subsequently, 10 ml of sterile water was added to each plate, and the mycelium was scraped with a glass spreader. After the fungal suspension was centrifuged at 13,800 × *g* for 1 min, the supernatant was removed, and the pellet was resuspended in sterile water with a weight 9-fold that of the pellet. The bacterial pathogen was cultured in LB liquid medium at 37°C for 24 h. Bacterial cells were harvested by centrifugation at 13,800 × *g* for 1 min and resuspended in sterile water to an optical density of 1.00 at 600 nm. Afterward, 0.1 ml of the fungal suspension (100-fold dilution) was spread evenly on PDA plates, while 0.1 ml of bacterial suspension (1,000-fold dilution) was spread evenly on beef extract peptone agar in petri dishes. Three sterilized Oxford cups were placed in each plate, and each cup was inoculated with 0.25 ml of cell-free supernatant of *B. amyloliquefaciens* Ba13. The inoculated plates were kept in a 4°C refrigerator for 24 h of diffusion and then cultured upside down at 30°C for 2 to 3 days. The diameter of the growth inhibition zone was measured to evaluate the antagonistic effects of strain Ba13 on pathogens.

### Phylogenetic and comparative genomic analyses.

TBtools (71) was used to extract the 16S rRNA gene and DNA topoisomerase subunit B (*gyrB*) gene from the sequencing results of strain Ba13. BLAST comparison of the extracted gene sequences was performed in the NCBI database. The whole genomes of strains with the highest similarity ranking, other species with close evolutionary distance, and three common species under the genus *Bacillus* were downloaded (*n *= 24; Table S1 in the supplemental material), from which the *gyrB* gene sequences were extracted. Muscle alignment was performed using Mega X, and the neighbor-joining method was used to construct a phylogenetic tree (72). The whole-genome data of strains *B. velezensis* FZB42 (accession NC_009725), *B. velezensis* SQR9 (accession CP006890), B. subtilis subsp*. subtilis* strain168 (accession CP052842), and *B. amyloliquefaciens* DSM7 (accession FN597644) were also downloaded from the NCBI database. The whole genome of strain Ba13 was used as the reference genome for comparative genomic analysis using BRIG v0.95 (73), and the results were used to draw a comparative genomic map.

### Prediction of secondary metabolites and secretory proteins.

Gene clusters of secondary metabolites in the genome of *B. amyloliquefaciens* Ba13 were predicted using antiSMASH v6.0.0 (74) and mapped using genelibs (https://www.genelibs.com/gb/pages/index.jsf). SignalP v5.0 (75) was used to predict the classical secretory proteins (signal peptides), and TMHMM v2.0 (76) was used to predict the transmembrane helix structure and secretion type. Phobius (77) was also used to predict signal peptides and transmembrane helix structures. Subsequently, ProtCompb v9.0 (Softberry, Mount Kisco, NY, USA) was used for subcellular localization, TatP v1.0 (78) was used to predict the TAT signal domain, and LipoP v1.0 (79) was used to predict the recognition sites of lipoprotein signal peptidase (SP II). Based on the prediction results, the proteins with transmembrane structures outside the signal peptide region were classified as transmembrane proteins, and the proteins with a TAT motif were defined as exogenous proteins secreted by the TAT secretion system. Other proteins located in cells were classified as intracellular proteins. Proteins with SP II recognition sites were considered membrane surface proteins, and the remaining proteins were considered classic secretory proteins secreted by the Sec system.

### Detection of plant hormones and synthetic intermediates.

For qualitative detection, *B. amyloliquefaciens* Ba13 was cultured in beef extract peptone broth supplemented with 5 mM l-TRP (30) at 180 rpm and 30°C for 72 h. After centrifugation at 13,800 × *g*, 2 ml of the supernatant was mixed with an equal volume of Pilet and Chollet (PC) reagent (12 g of FeCl_3_ per liter in 7.9 M H_2_SO_4_) and placed in the dark at 25°C for 30 min (80). The mixture was centrifuged again at 13,800 × *g* for 1 min, and the solution color was observed with a red color indicating the presence of IAA. For quantitative testing, strain Ba13 was cultured in beef extract peptone broth at 30°C and 180 rpm for 48 h and then incubated at low temperature for cooling. The cell-free supernatant was obtained by filtration through a 0.22-μm microporous membrane and then extracted with methanol/water/formic acid solution (15:4:1 [vol/vol/vol]). After concentration, the extract was redissolved in 80% methanol/water solution, filtered through a 0.22-μm microporous membrane, and then placed in a sample vial for quantitative analysis by LC-MS/MS (81). The absolute contents of the target compounds were calculated by substituting the linear equation into the peak area of the chromatographic peak.

### Data availability.

The data sets generated during the current study are available in the NCBI repository. (https://www.ncbi.nlm.nih.gov/sra, accession number CP073635).
