# Supplementary material for: Genomic Analysis Reveals Potential Mechanisms Underlying Promotion of Tomato Plant Growth and Antagonism of Soilborne Pathogens by Bacillus amyloliquefaciens Ba13
Source: Microbiol Spectr. 2021 Nov 10;9(3):e01615-21. doi: 10.1128/Spectrum.01615-21 (PMC8579842; doi:10.1128/Spectrum.01615-21)
Supplement: SUPPLEMENTAL FILE 1 — Supplemental material. Download Spectrum.01615-21-s0001.pdf, PDF file, 0.7 MB [file spectrum.01615-21-s0001.pdf]

Genomic Analysis Reveals Potential Mechanisms Underlying Promotion of Tomato Plant Growth and Antagonism of Soil-borne Pathogens by *Bacillus amyloliquefaciens* Ba13

Chenglong Ji,[a] Meilin Zhang,[a] Zirong Kong,[a] Xue Chen,[a] Xing Wang,[a] Wei Ding,[b] Hangxian Lai,[a] Qiao Guo [a]

[a]College of Natural Resources and Environment, Northwest A&F University, Yangling, China

[b]Laboratory of Natural Products Pesticide, College of Plant Protection, Southwest University, Chongqing, China

Address correspondence to Hangxian Lai, laihangxian@163.com & Qiao Guo, shuiyiwei83@163.com

SUPPLEMENTAL MATERIAL

Genome extraction, sequencing, assembly and annotation. Total bacterial DNA was extracted from *B. amyloliquefaciens* Ba13 culture using a bacterial genomic DNA extraction kit (BioTeke Corp., Wuxi, China). The DNA concentration, purity, and integrity were evaluated using the Nanodrop One spectrophotometer (Nanodrop Technologies, Wilmington DE, USA), Qubit 3.0 Fluorometer (Life Technologies, Carlsbad, CA, USA), and 0.35% agarose gel electrophoresis. Whole-genome sequencing was performed using PromethION (Oxford Nanopore Technologies, Oxford, UK) and NovaSeq 6000 (Illumina, San Diego, CA, USA) sequencers. After filtering low-quality and excessively short reads, genome data were assembled using Unicycler v0.4.8 (1). The assembled whole-genome sequence was analyzed using Prokka v1.1.2 (2) and the genes predicted. Subsequently, the predicted gene sequences were compared against the COG, KEGG, Uniprot, and other databases, to obtain the annotation results. The complete genome sequence of *B. amyloliquefaciens* Ba13 was deposited in the NCBI database (https://www.ncbi.nlm.nih.gov/) (Accession number: CP073635).

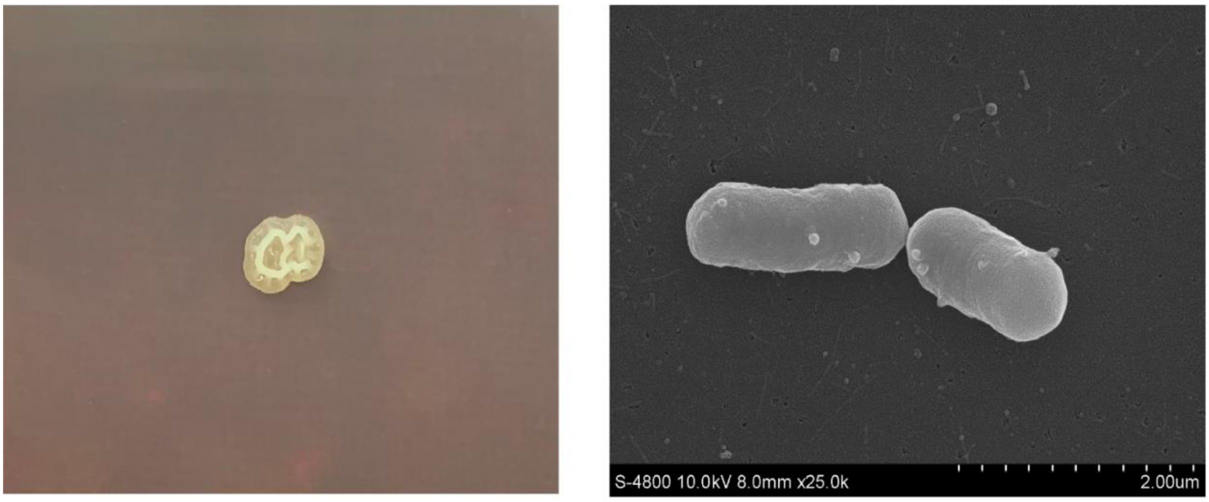

(a) (b)

Fig S1 Morphological characteristics of *B. amyloliquefaciens* Ba13. a) A colony formed on beef extract peptone agar plate. b) Cell morphology under scanning electron microscope.

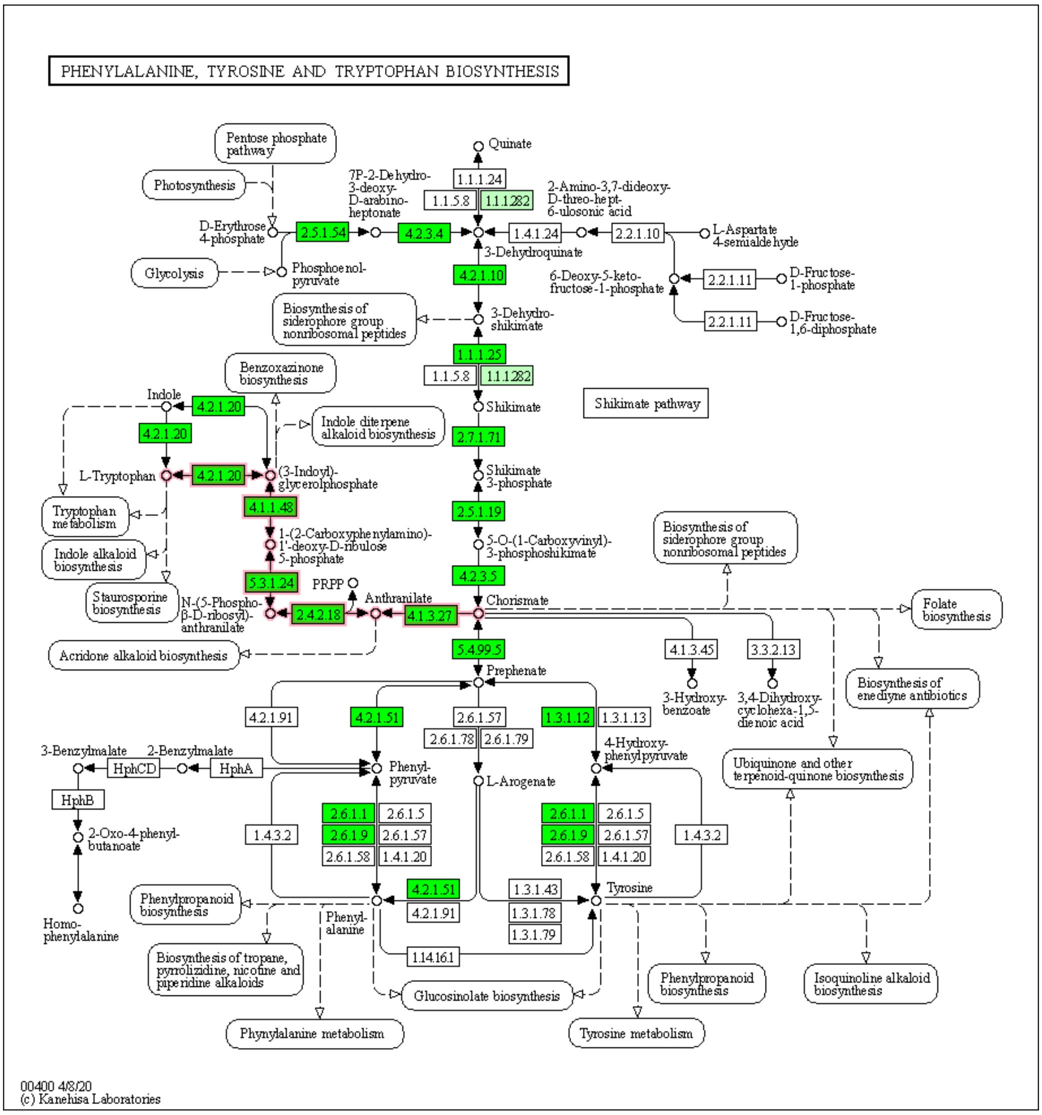

Fig S2 Genes of *B. amyloliquefaciens* Ba13 enriched in the tryptophan biosynthetic pathway (KO00400).

The bacterial genome contains a complete TRP and indole biosynthetic pathway.

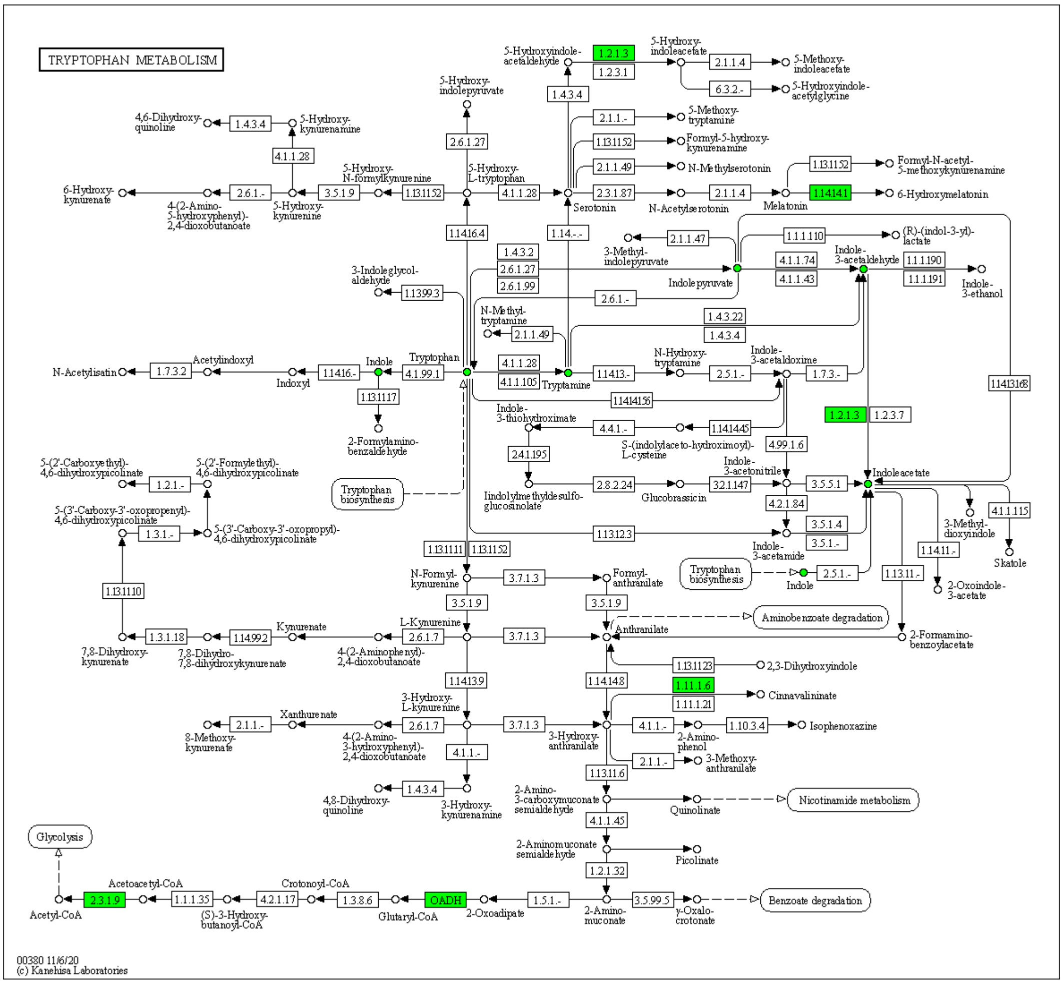

Fig S3 Genes of *B. amyloliquefaciens* Ba13 enriched in the tryptophan metabolic pathway (KO00380). A complete IAA biosynthetic pathway is lacking.

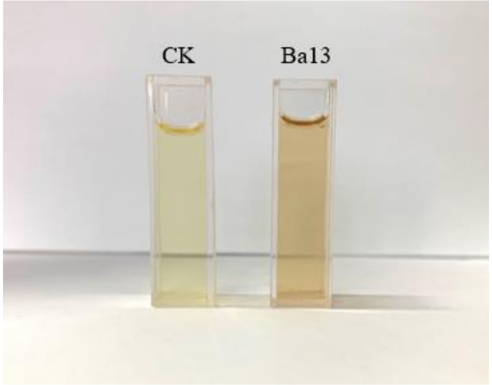

Fig S4 Detection of indole-3-acetic acid produced by *B. amyloliquefaciens Ba13*. The culture supernatant of strain Ba13 was mixed with reagent PC for color reaction. CK is the control (culture medium without bacterial inoculation), and Ba13 is the bacterial treatment (48-h-old culture supernatant of strain Ba13).

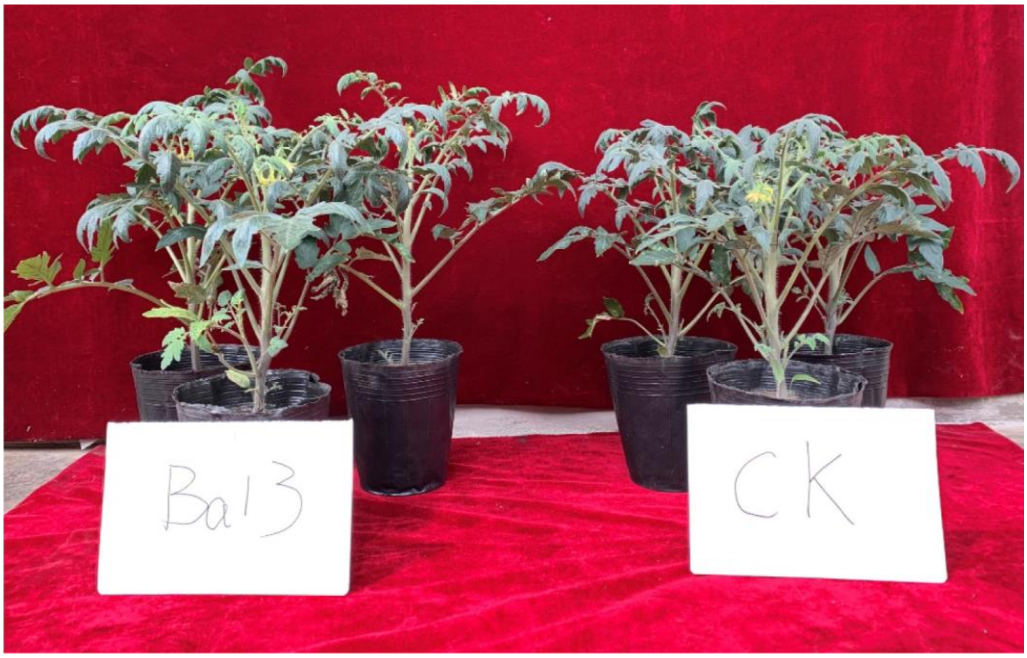

Fig S5 A photograph showing the growth of tomato plants grown on loessial soil treated with strain Ba13 (Ba13) and without strain Ba13 (CK) on day 21.

REFERENCES

1. Wick RR, Judd LM, Gorrie CL, Holt KE. 2017. Unicycler: Resolving bacterial genome assemblies from short and long sequencing reads. PLoS Comput Biol 13:e1005595.
2. Seemann T. 2014. Prokka: rapid prokaryotic genome annotation. Bioinformatics 30:2068-9.
